# Phosphoproteome data from abscisic acid and ethylene treated *Glycine max* leaves

**DOI:** 10.1016/j.dib.2018.08.037

**Published:** 2018-08-21

**Authors:** Ravi Gupta, Cheol Woo Min, Qingfeng Meng, Tae Hwan Jun, Ganesh Kumar Agrawal, Randeep Rakwal, Sun Tae Kim

**Affiliations:** a Department of Plant Bioscience, Life and Industry Convergence Research Institute, Pusan National University, Miryang 50463, South Korea; b Research Laboratory for Biotechnology and Biochemistry (RLABB), GPO Box 13265, Kathmandu, Nepal; c GRADE Academy Private Limited, Adarsh Nagar-13, Birgunj, Nepal; d Faculty of Health and Sport Sciences and Tsukuba International Academy for Sport Studies (TIAS), University of Tsukuba, 1-1-1 Tennodai, Tsukuba, Ibaraki 305-8577, Japan; e Global Research Center for Innovative Life Science, Peptide Drug Innovation, School of Pharmacy and Pharmaceutical Sciences, Hoshi University, 4-41 Ebara 2-chome, Shinagawa, Tokyo 142-8501, Japan

## Abstract

The data reported here are associated with the article “Comparative phosphoproteome analysis upon ethylene and abscisic acid treatment in *Glycine max* leaves” [1]. Phosphorylation plays a critical role in the regulation of the biological activities of proteins. However, phosphorylation-mediated regulation of proteins and pathways involved in ethylene (ET) and abscisic acid (ABA) signaling is currently poorly understood. Therefore, we used a shotgun proteomics approach to identify the phosphopeptides and phosphoproteins in response to ET, ABA and combined ET+ABA treatments. Here, we present the Mass spectrometry, protein–protein interaction, Gene ontology and KEGG data associated with the ET and ABA signaling in soybean leaves [1].

## Specifications Table

**Table:** 

Subject area	Biology
More specific subject area	Plant Science, Phosphoproteomics, Plant hormones
Type of data	Tables and figures
How data was acquired	Mass spectrometer, and UHPLC Dionex UltiMate^®^ 3000 (Thermo Fisher Scientific, USA) system coupled with QExactive™ Orbitrap High-Resolution Mass Spectrometer (Thermo Fisher Scientific, USA)
Data format	Raw, analyzed
Experimental factors	ABA and ET treatments, TiO_2_ based enrichment of phosphopeptides
Experimental features	ABA and ET induced changes in phosphoproteome were analyzed
Data source location	Department of Plant Bioscience, College of Natural Resources and Life Science, Pusan National University at Miryang, South Korea (latitude 35N)
Data accessibility	Data are within this article
Related research article	R. Gupta, C.W. Min, Q. Meng, G.K. Agrawal, R. Rakwal, S.T. Kim, Comparative Phosphoproteome Analysis upon Ethylene and Abscisic acid Treatment in Glycine max Leaves, Plant Physiol. Biochem. (2018) [1].

## Value of the data

• This dataset provides information about the 802 phosphopeptides, identified phosphosites and associated upstream kinase(s).
• The identified phosphopeptides, phosphosites and enriched motifs further supplement the known phosphoproteome map of soybean.
• Moreover, results reported here enhance our understanding of phosphorylation mediated regulation of ABA and ET responses to soybean leaves.

## 1 Data

**Fig. 1:**
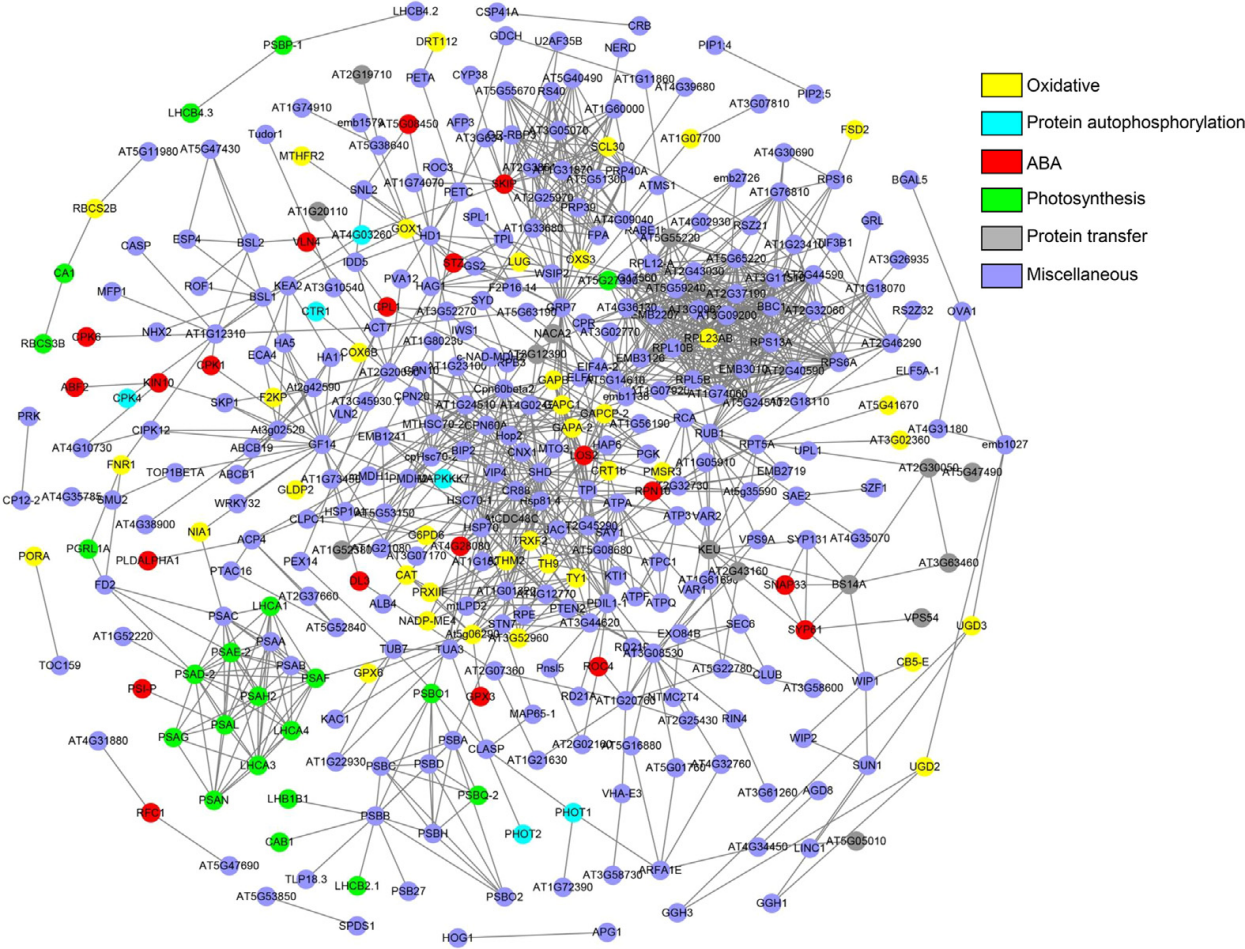
Protein–protein interaction network of the all the identified phosphoproteins using Cytoscape integrated with STRING. Functional annotation of the identified proteins was carried out using Gene Ontology database.

**Fig. 2:**
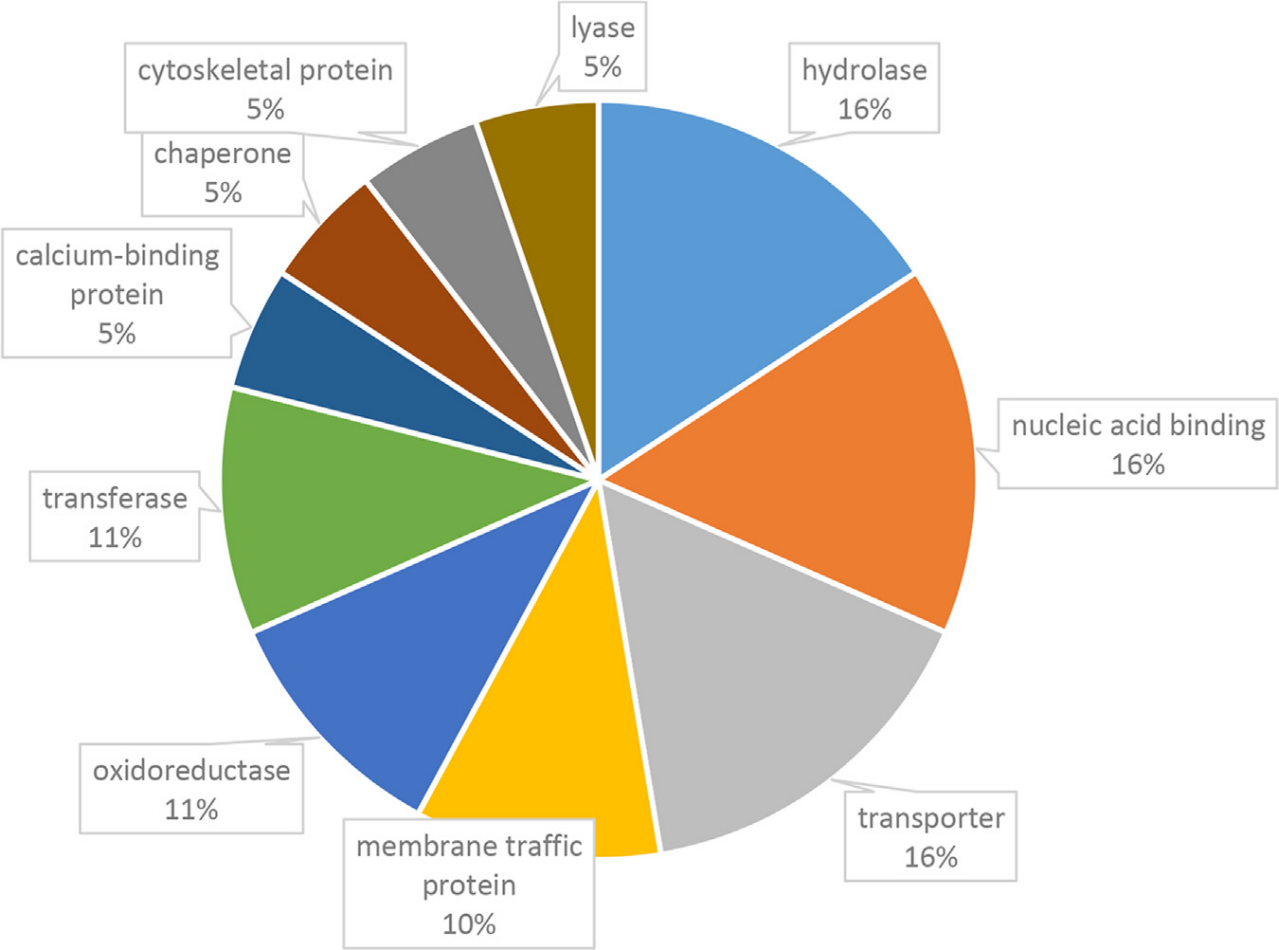
PANTHER-protein class classification of phosphoproteins showing a significant change in phosphosite intensity in response to phytohormone treatment.

Figures reported here depict the interaction network obtained from the identified phosphoproteins (Fig. 1), and functional annotation of the ABA and ET modulated phosphoproteins (Fig. 2) from soybean leaves. Supplementary tables show the list of the identified phosphoproteins (Supplementary Table 1), and the table for the protein–protein interaction (Supplementary Tables 2 and 3). Detailed description of the data and methods is reported previously [1].

## 2 Experimental design, materials and methods

### 2.1 Plant growth conditions and hormone treatments

*Glycine max* cv. Daewon seeds were germinated in the soil and allowed to grow in a growth chamber at 25 °C (16/8 h day/light cycle, 70% relative humidity) for one month [2]. Ethephon and ABA treatments were given as described previously [1], [2] and leaves were harvested after 3 h for phosphoproteome analysis.

### 2.2 Protein extraction and phosphopeptide enrichment

Protein extraction and phosphopeptides enrichment were carried out as described previously [1]. In brief, 1 g of leaves were homogenized in 5 mL RIPA buffer containing phosSTOP phosphatase inhibitor cocktail (Roche, Basel, Switzerland) and protease inhibitor cocktail (Thermo Fisher Scientific, USA) and centrifuged at 12,000 rpm for 20 min at 4 °C. The supernatant thus obtained was subjected to methanol-chloroform precipitation and the obtained pellets were solubilized in 1× SDS-loading buffer or 6 M urea for SDS-PAGE or in-solution trypsin digestion respectively. One milligram of protein from each sample was used for in-solution trypsin digestion [3] and phosphopeptides enrichment was carried out using TiO_2_ based phosphopeptide enrichment kit (Pierce Biotechnology) following manufacturer׳s protocol.

### 2.3 Phosphopeptide identification and data processing

QExactive™ Orbitrap High-Resolution Mass Spectrometer (Thermo Fisher Scientific, USA) was used for the identification of enriched phosphopeptides exactly using the same protocol as described earlier [4]. Data analysis was carried out using MaxQuant software [5] v.1.5.0.0 using Andromeda as a search engine [6] and downstream data processing was carried out using Perseus software [7]. Phosphopeptides that were reproducibly identified in at least two out of three replicates of at least one sample with score > 40 and delta score > 7 were considered as valid identification and used for the further analysis [1].

### 2.4 Functional annotation of the identified proteins

DAVID functional annotation tool (https://david.ncifcrf.gov/tools.jsp) with integrated PANTHER Gene Ontology (GO) and KEGG pathways was used for the functional annotation of the identified phosphoproteins. For the construction of protein–protein interaction (PPI) network, homologs of the identified phosphoproteins were searched in the Arabidopsis database obtained from Phytozome (https://phytozome.jgi.doe.gov/pz/portal.html). The obtained Arabidopsis homologs were used for the PPI analysis by the Search Tool for Retrieval of Interacting Genes/Proteins (STRING) database (http://string-db.org/) and arranged using Cytoscape tool.

## Appendix A Supplementary material

Supplementary material **Supplementary Table 1.** List of the identified phosphopeptides in response to ABA, ET and combined ABA+ET treatment in soybean leaves.

Supplementary material **Supplementary Table 2.** Protein–protein interaction network constructed in this work using all the identified phosphoproteins.

Supplementary material **Supplementary Table 3.** Protein–protein interaction network constructed using proteins involved in ABA signaling, oxidative, photosynthesis, protein autophosphorylation and protein transport.
